# 364. Novel Polygalacturonic and Caprylic Acid (PG+CAP) Antimicrobial Wound Ointment is Effective in Managing Microbially Contaminated Chronic Wounds in a Pilot Prospective Randomized Clinical Study

**DOI:** 10.1093/ofid/ofad500.434

**Published:** 2023-11-27

**Authors:** Ray Y Hachem, Christopher Hakim, Hiba Dagher, Roula Samaha, Diya' Hammoudeh, Nelson Hamerschlak, Janane Nasr, Joel Rosenblatt, Ying Jiang, Anne-Marie Chaftari, Odette Ghanem, Amir Ibrahim, Abdul Rahman Bizri, Issam I Raad

**Affiliations:** MD Anderson UT, Houston, Texas; University of Beirut, Beirut, Beqaa, Lebanon; UT MD Anderson Cancer Center, Houston, Texas; Lebanese American University, Beirut, Beqaa, Lebanon; American University of Beirut, Beirut, Beqaa, Lebanon; Hospital Israelita Albert Einstein, São Paulo, Brasil, Sao Paulo, Not Applicable, British Indian Ocean Territory; American University of Beirut, Beirut, Beqaa, Lebanon; MD Anderson UT, Houston, Texas; The University of Texas MD Anderson Cancer Center, Houston, Texas; MD Anderson UT, Houston, Texas; American University of Beirut, Beirut, Beqaa, Lebanon; American University of Beirut, Beirut, Beqaa, Lebanon; American University of Beirut, Beirut, Beqaa, Lebanon; MD Anderson UT, Houston, Texas

## Abstract

**Background:**

The use of antibiotics and antiseptics in the management of biofilm colonized chronic wounds is not fully effective in eradicating multidrug-resistant organisms. We developed PG+CAP ointment combining naturally derived agents commonly used in skin care and showed in our in vitro study that PG-+CAP combination ointment could rapidly eradicate gram positive, gram negative and fungal organisms embedded in biofilm within 1 hour. Furthermore, in a full thickness, microbially contaminated swine wound model study we found that with daily ointment application, PG+CAP ointment was significantly better at healing wounds at 2 weeks compared to MediHoney and was also significantly better at eradicating microbes colonizing wound tissues. In this current pilot human study, we compared PG+CAP ointment to MediHoney at three international centers.

**Methods:**

This is a multicenter prospective randomized open label comparative study where patients with chronic full-thickness wounds at three academic international medical centers (American University of Beirut and Lebanese American University, Lebanon and Albert Einstein Medical Center, Brazil) were randomized to a daily treatment with either PG+CAP ointment or MediHoney. Clinical and wound assessments were obtained weekly up to 6 weeks. Full improvement was defined as 50% or greater decrease in wound size and/or a 75% or greater re-epithelialization and granulation of the wound with no necrosis or related adverse events (AEs) or sepsis. The validated Pressure Ulcer Scale for Healing (PUSH) score was used to track wound healing.

**Results:**

A total of 20 patients with chronic wounds were included (10 patients in each arm). Age and clinical characteristics were comparable in both groups. Diabetes mellitus tended to occur more frequently in the PG+CAP arm (See Table 1-P=0.06). Full improvement/healing occurred in all patients (100%) randomized to PG+CAP with no AEs vs 70% full improvement in the MediHoney arm. Patients who received PG+CAP had more than two-fold wound improvement in terms of their PUSH scores compared to MediHoney:

**Table 1.**
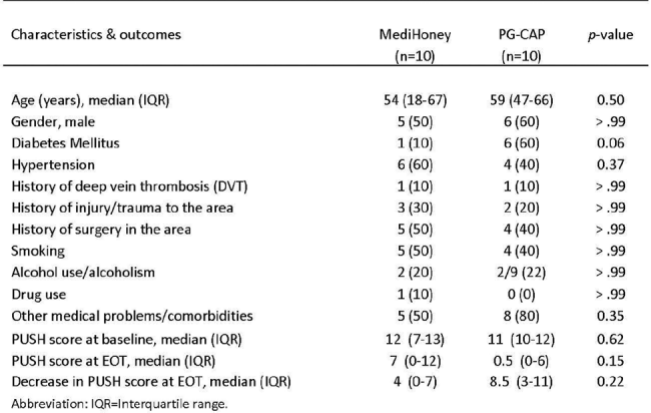
Characteristics & Outcomes

**Conclusion:**

Based on preliminary data from this prospective randomized multicenter trial, PG+CAP is safe and possibly more effective in achieving full improvement of chronic contaminated wounds when compared to MediHoney.

**Disclosures:**

**Joel Rosenblatt, PhD**, Novel Anti-Infective Technologies, LLC: Licensed Technology **Issam I. Raad, Distinguished Professor**, Novel Anti-Infective Technologies, LLC: Technology License

